# Activation of Insulin Gene Expression via Transfection of a CRISPR/dCas9a System Using Magnetic Peptide-Imprinted Nanoparticles

**DOI:** 10.3390/pharmaceutics15041311

**Published:** 2023-04-21

**Authors:** Mei-Hwa Lee, James L. Thomas, Chien-Yu Lin, Yi-Chen Ethan Li, Hung-Yin Lin

**Affiliations:** 1 Department of Materials Science and Engineering, I-Shou University, Kaohsiung 84001, Taiwan; 2 Department of Physics and Astronomy, University of New Mexico, Albuquerque, NM 87131, USA; 3 Department of Chemical and Materials Engineering, National University of Kaohsiung, Kaohsiung 81148, Taiwan; 4 Department of Chemical Engineering, Feng Chia University, Taichung 40724, Taiwan

**Keywords:** insulin, gene expression, CRISPR/dCas9a, gene activation, molecular imprinting

## Abstract

A CRISPRa transcription activation system was used to upregulate insulin expression in HEK293T cells. To increase the delivery of the targeted CRISPR/dCas9a, magnetic chitosan nanoparticles, imprinted with a peptide from the Cas9 protein, were developed, characterized, and then bound to dCas9a that was complexed with a guide RNA (gRNA). The adsorption of dCas9 proteins conjugated with activators (SunTag, VPR, and p300) to the nanoparticles was monitored using both ELISA kits and Cas9 staining. Finally, the nanoparticles were used to deliver dCas9a that was complexed with a synthetic gRNA into HEK293T cells to activate their insulin gene expression. Delivery and gene expression were examined using quantitative real-time polymerase chain reaction (qRT-PCR) and staining of insulin. Finally, the long-term release of insulin and the cellular pathway related to stimulation by glucose were also investigated.

## 1. Introduction

Insulin is an anabolic hormone that is produced by the beta-cells of the pancreatic islets in the human body [1]. Insulin promotes glucose uptake and metabolism, reducing blood sugar levels [2]. A reduction or absence of insulin activity causes diabetes mellitus (DM), which is a condition that is characterized by a high blood sugar level, or hyperglycemia [3]. Type 1 diabetes refers to failure of the pancreas to produce enough insulin for the reduction of blood glucose owing to a loss of beta-cells [4,5,6]. The pathogenesis of type 2 diabetes is not well understood, but a reduced population of islet beta-cells, the weakened secretory function of islet beta-cells, and the insulin-resistance of peripheral tissue are known to be involved [7,8,9]. Many diabetes mellitus patients require insulin to keep blood glucose levels in a healthy range [10]. Various insulin delivery techniques, involving vials and syringes, insulin pens, and insulin pumps, have been reviewed [10], but the development of new therapeutics depends on a better understanding of the long-term release of insulin.

In the last decade, the CRISPR/Cas9 system has been exploited in gene-editing applications, by using a mutated, inactive (“dead”) Cas9, dCas9, for the activation or repression of gene expression. The dCas9 protein exhibits no endonuclease activity, but retains its ability to bind a guide RNA (gRNA), which targets the dCas9 and associated transcription activators, to the desired DNA sequence [11]. Many dCas9 activator systems have been examined to determine their capacity to induce robust gene expression [12]. The cellular delivery of the CRISPR/Cas9 system [13] are transfection with (1) a DNA plasmid that encodes both the Cas9 protein and the guide RNA [14], (2) Cas9 mRNA and a separate gRNA [15], and (3) a ribonucleoprotein (RNP) complex, comprising Cas9 protein with a gRNA [16,17,18]. The mechanism of gene activation using CRISPR technology is described elsewhere [19]; briefly, Cas9-based transactivator is targeted to DNA sequences by guide RNA molecules. Coexpression of this transactivator and combinations of guide RNAs in human cells induces specific expression of endogenous target genes [19].

Molecularly imprinted polymers (MIPs) are tailor-made recognition polymers that are used in biosensing [20,21], bioseparation [22], and delivery [23,24,25]. Ansari and Masoum reviewed protein-imprinting methods, the rational design of protein-MIPs, metrics of the success of protein imprinting, and imprinted protein biomarkers [26], focusing on MIP particles that can mimic biological receptors and sensitively and selectively recognize specific target molecules. The delivery of a CRISPR/Cas9 gene-editing system for in vitro genome editing [27], along with related therapeutic applications [16] and challenges [28], has also been recently reviewed. Delivery methods include high-pressure injection [26], as well as complexation with polymers, lipids, and even with a bacterial pore-forming toxin [26,29]. Carboxymethyl chitosan has been used with biotinylated AS1411 ligand to deliver the CRISPR-Cas9 plasmid to knock out the CDK11 protein gene, and the consequent reduction of the amount of this protein downregulated the development of cancer cells [30]. PEGylated chitosan has also been used as a non-viral aerosol for the mucosal delivery of the CRISPR/Cas9 system, demonstrated in vitro [31]. Our earlier work demonstrated multigene activation by the delivery of CRISPR/dCas9 ribonucleoproteins using magnetic peptide-imprinted chitosan nanoparticles (MPIPs) for cellular reprogramming [24]. Using magnetic chitosan polymers (MMIPs) for transfection has important advantages: (1) the magnetic particles facilitate manipulation and separation, using magnetic fields [32], and (2) chitosan enhances cellular transfection [24]. In the previous study, a conventional vector including plasmid and lipofectamine were employed for activating insulin by CRISPR/Cas9a in human cells. Moreover, the activation of the INS gene was sustained for at least 21 days post transfection, which was assessed by RT-PCR post transfection in the CRISPR-on systems with all of the sgRNA in the HEK293T cells [33]. In this work, the gRNA of insulin was synthesized and used to form ribonucleoproteins (RNPs) with dCas9-VPR, which were then bound on dCas9-recognizing MPIP nanocomposites [34]. The MPIPs were used to transfect HEK293T cells, endowing them with the capability for long-term glucose-stimulated insulin secretion. This provides a novel model system in which to study long-term insulin release, as well as elucidating new therapeutic strategies.

## 2. Experiment

**Scheme 1:**
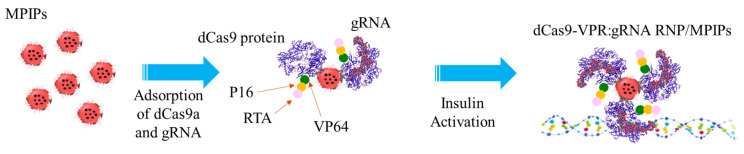
Activation of insulin gene expression via transfection of a CRISPR/dCas9a system using magnetic peptide-imprinted nanoparticles.

The experimental details of the synthesis of the gRNA for insulin and that of the nanocomposites can be found in the supplemental information (Scheme 1).

### 2.1. Delivery of RNP with MPIPs to HEK 293T Cells

RNPs that contained dCas9-VPR proteins and gRNA on MPIPs were used to activate the transcription of insulin in HEK 293T cells. The target sequence of Edit-R CRISPRa crRNA for Human INS (Dharmacon, Inc., Lafayette, CO, USA) is GCGGCAGGGGTTGAGAGGTA. Our previous work demonstrated that using magnetic nanoparticles can provide for easier manipulation [20] for the extraction of dCas9 protein from cellular lysate [32]. Moreover, the use of magnetic nanoparticles in this work is also useful for the precise delivery of RNPs [24]. The preparation of MPIP can be found in the supplemental information [24,32]. Briefly, gRNAs composed of equal amounts of 1.0 μL crRNAs and tracrRNA at 10 μM were mixed at room temperature for 30 min. Then, 2 μL of dCas9-VPR proteins at 100 ng/mL were loaded with the above gRNAs for 30 min at room temperature to form RNPs. Various concentrations of RNPs were then immobilized with 100 μg of MPIPs by the epitope recognition of Cas9 proteins [24] for 30 min at room temperature (ca. 25 °C). The MPIPs were then removed after 24 h of incubation. The same amounts of RNPs were added to Lipofectamine™ CRISPRMAX™ Cas9 transfection reagent, based on the protocol (https://assets.thermofisher.com/TFS-Assets%2FLSG%2Fmanuals%2FMAN0014545_lipofectamine_crispermax_QR.pdf, accessed on 6 April 2023). High concentrations of nanoparticles may lower the cellular viability [32]. Therefore, the optimal nanoparticle concentration was employed with varying RNP concentrations.

### 2.2. Measurement of Insulin Release with Glucose Stimulation

Culture medium was removed and cells were washed with PBS buffer (#70011069 Gibco), with added DMEM medium (without glucose, #11966, Gibco (Thermo Fisher Scientific Inc., Waltham, MA, USA)) for incubation for an hour for the simulation before intake. The medium was replaced with medium containing 5 mg/mL glucose and incubated with cells for an hour at 37 °C to simulate the intake, and the medium (500 μL/well) was then replaced with normal medium for subculture about every three days. These media were collected for measurement of the concentration of insulin with a Human Insulin ELISA Kit (#KAQ1251, Thermo Fisher Scientific, Waltham, MA, USA). The protocol can be found from the supplier’s website (https://www.thermofisher.com/document-connect/document-connect.html?url=https://assets.thermofisher.com/TFS-Assets%2FLSG%2Fmanuals%2FMAN0004817_KAQ1251_PI.pdf, accessed on 6 April 2023). The same protocol was employed for the measurements of insulin concentrations on various days after transfection and stimulation.

## 3. Results and Discussion

**Figure 1:**
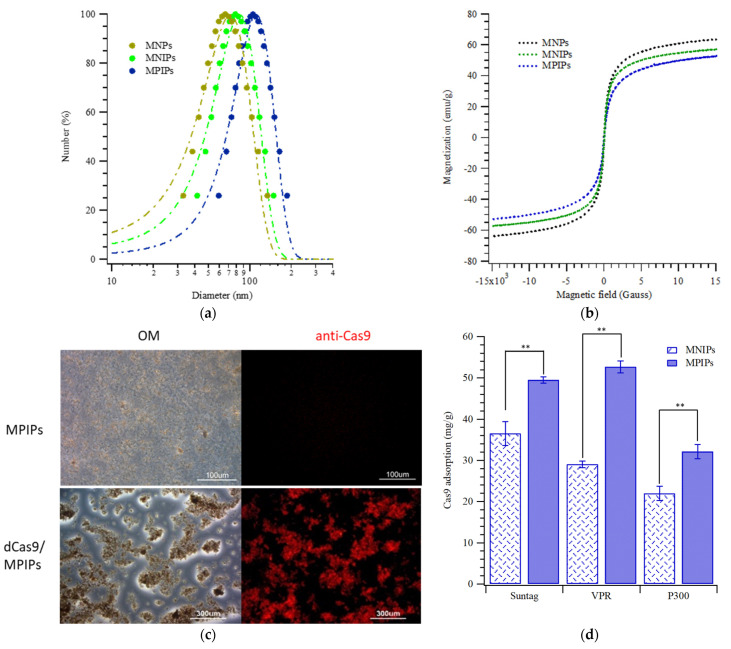
(**a**) Size distribution of the magnetic nanoparticles (MNPs), magnetic non-imprinted chitosan nanoparticles (MNIPs), and peptide-imprinted chitosan nanoparticles (MPIPs). (**b**) Magnetization curves of MNPs, MNIPs, and MPIPs after template removal. (**c**) Immunostaining images of MPIPs without and with adsorption of dCas9a proteins. (**d**) Adsorption capacities of MPIPs with the same concentration of dCas9a proteins. All experiments were carried out in triplicate, and data are expressed as means ± standard deviation. Standard deviation is based on at least three measurements, and **: *p* < 0.005.

Figure 1 shows the characteristics of the magnetic peptide-imprinted chitosan nanoparticles (MPIPs); the peptide sequence is in the supporting information. Figure 1a shows the size distributions of magnetic nanoparticles (MNPs) and non-imprinted (MNIP) and imprinted (MPIP) composite nanoparticles, whose sizes after template removal are 72 ± 10, 78 ± 6, and 107 ± 18 nm (mean ± standard deviation), respectively. Figure 1b presents the magnetization of MNPs, MNIPs, and MPIPs after template removal; their saturated magnetizations were 71.4 ± 0.3, 63.3 ± 0.3, and 56.8 ± 0.3 emu/g, respectively. The saturated magnetization followed the order MNPs > MNIPs > MPIPs owing to the effective “dilution” of magnetization upon the addition of chitosan and template. (The buried template molecules can never be removed.) The inset in Figure 1b also shows the suspension and adsorption of MPIPs on the walls of a vial in a magnetic field. MPIPs were incubated with a relatively high concentration (3.0 mg/mL) of dCas9-VPR for visualization, and then separated by applying a magnetic field. Figure 1c presents the optical and immunohistochemical (IHC) staining of Cas9 proteins bound to MPIPs and merged images thereof. Figure 1d displays the adsorption of dCas9 that was conjugated with various activators such as Suntag, VPR, and P300 on the composite nanoparticles. The adsorption capacities of the extracted chimeric proteins were measured using ELISA kits. The adsorption capacities of MNIPs and MPIPs were found to be 29.0 ± 0.8 and 52.7 ± 1.4 mg/g (ca. 479 ± 13 nmol/g), respectively, for dCas9-VPR with molecular weight of ~220 kDa. Thus, the MPIPs, owing to their imprinting with a dCas9 peptide, are able to bind much more of the dCas9-VPR. This recognition is mediated by non-covalent interactions between the target peptide sequence and the imprinted chitosan. These figures and binding results reveal that the adsorbed dCas9 proteins can be stained with anti-Cas9 antibodies and that these dCas9 proteins can be adsorbed on the surface of MPIPs for the loading of gRNA. Figure S1a shows that the size distributions of MPIP with dCas9-VPR or RNPs at 20 nM are 171 ± 4 and 187 ± 3 nm, respectively. These large sizes likely reflect aggregation of a few nanoparticles. Figure S1b shows that cells treated with the dCas9-gRNA/MPIPs complex retained high viability (the apparent increase in “viability” reflects normal proliferation during the first three days), and thus that these nanoparticle delivery vehicles are biocompatible (at least in tissue culture [24].)

**Figure 2:**
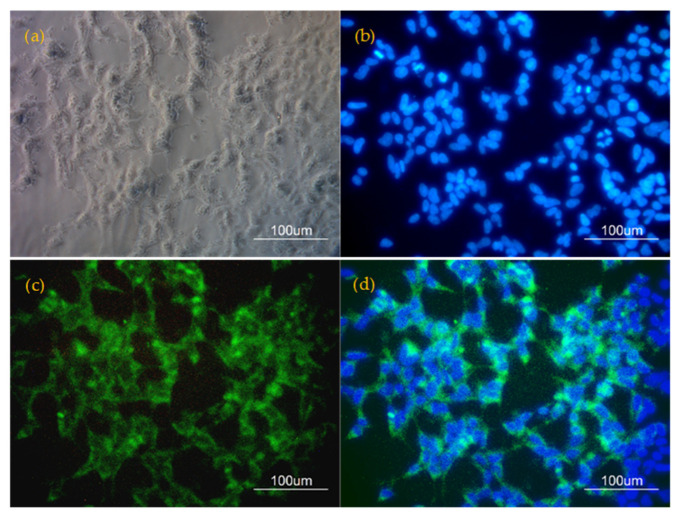
Immunohistochemistry (IHC) images of (**a**) optical, (**b**) DAPI-staining, (**c**) anti-insulin staining, and (**d**) merge of (**b**) and (**c**) in HEK-293T cells treated with MPIPs/dCas9-VPR and crRNA.

**Figure 3:**
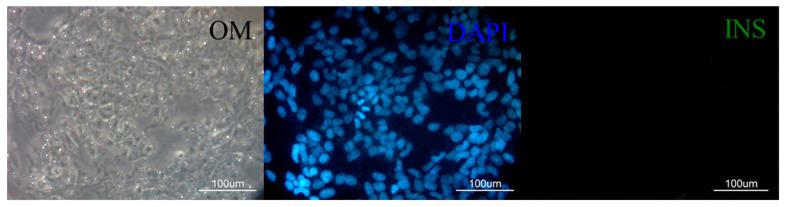
IHC images of optical, DAPI-staining, and anti-insulin staining in HEK-293T cells without RNPs/MPIPs.

Figure 2 and Figure 3 present optical, DAPI-, and insulin-immunostained images of fixed, permeabilized cells. Figure 2 images show localization of insulin in cells, which are clearly differentiated from the control results in Figure 3. Merged images are also shown, and the expressed insulin fills the cell cytoplasm, as seen in Figure 2d.

**Figure 4:**
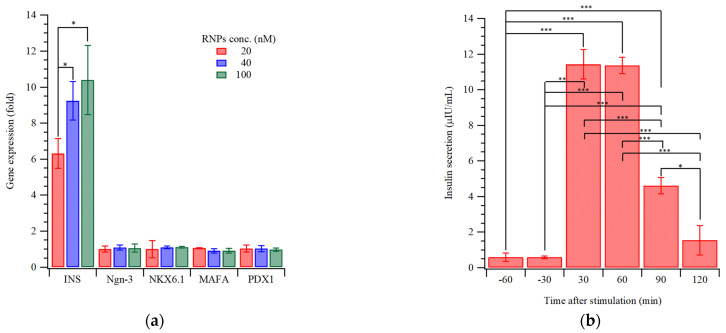

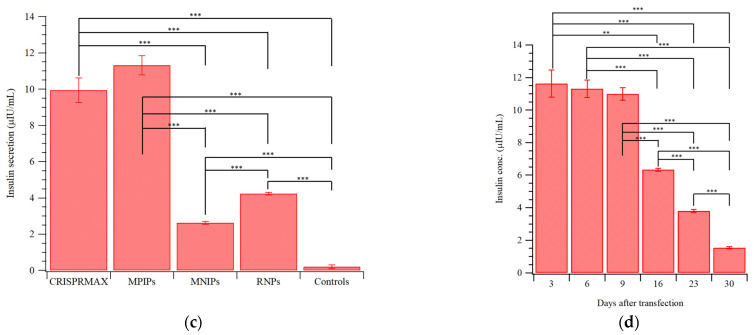
(**a**) Relative gene expression of key cellular differentiation genes of β-cells for activation of insulin with MPIPs/RNPs in HEK-293T cells. The administration of RNPs had no effect on the expression levels of these genes. (**b**) Insulin release from transfected HEK293T cells during an hour of glucose stimulation. (**c**) Insulin secretion of controls, RNPs, with MNIP, MPIPs, or Lipofectamine™ CRISPRMAX™-treated HEK293T cells after high glucose stimulation. (**d**) Insulin secretion 30 min after glucose re-stimulation on the 3rd, 6th, 9th 16th, 23rd, and 30th day. (*: *p* < 0.05, **: *p* < 0.005, and ***: *p* < 0.0005) The image in (**b**) has been published elsewhere [34], where it was used only for comparison purposes. Reproduced from Ref. [34] with permission from the Royal Society of Chemistry.

**Figure 5:**
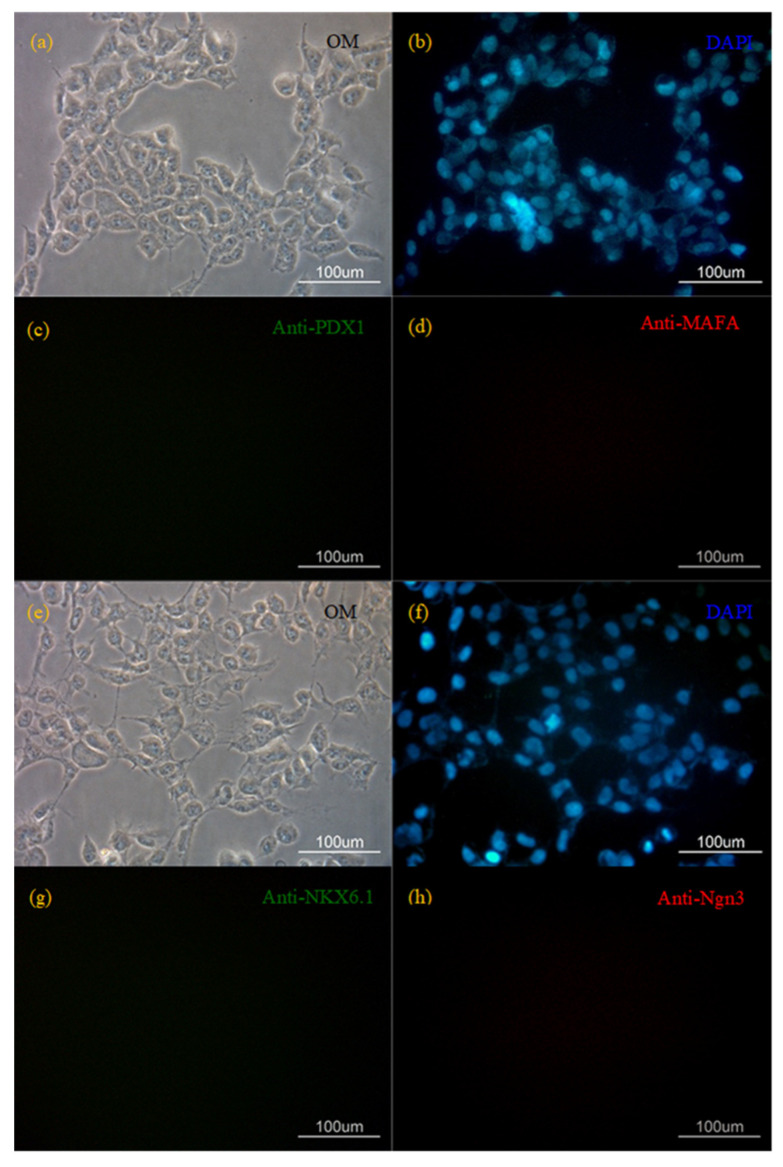
Immunohistochemistry (IHC) images of (**a**,**e**) optical, (**b**,**f**) DAPI, (**c**) anti-PDX1, (**d**) anti-MAFA, (**g**) anti-NKX6.1, and (**h**) anti-Ngn3 staining in HEK293T cells treated with MPIPs/RNPs.

The differentiation to pancreatic β-cells is mainly regulated by the transcription factors Neurog3 (Ngn-3), NK6 Homeobox 1 (NKX6.1), MAFA, and pancreatic and duodenal homeobox 1 (PDX1) [35]. Therefore, the expressions of insulin mRNA, as well as these genes, were measured following the administration of MPIPs/RNPs, shown in Figure 4a. The administration of MPIPs/RNPs to HEK293T cells “short-circuits” the need for these transcription factors to be upregulated for insulin production. The expression of insulin mRNA in HEK293T cells with 20, 40, and 100 nM RNPs is approximately six-, nine-, and ten-fold higher, respectively, than that of the controls. The immunostaining of other transcription factors (Ngn-3, NKX6.1, MAFA, and PDX1) demonstrated low expression, as shown in Figure 5. Significantly, stimulation of transfected HEK 293T cells with glucose stimulates insulin release. The RNP concentration at 20 nM is then selected for the latter glucose stimulation measurements. Figure 4b displays the release of insulin after stimulation with 5.0 mg/mL of glucose for an hour; after 30 min, the insulin concentration had reached 11.4 ± 0.8 μIU/mL, which was maintained for 30 min, before gradually decreasing over the following hour. Figure 4c presents the insulin secretion of controls, RNPs, with MNIPs, MPIPs, or Lipofectamine™ CRISPRMAX™-treated HEK293T cells after high glucose stimulation for 30 min. Interestingly, the free RNPs yield approximately 4.2 ± 0.1 μIU/mL of insulin after six days of transfection and an hour of high glucose stimulation. The insulin concentrations with MNIPs and MPIPs as carriers were about 2.6 ± 0.1 and 11.3 ± 0.5 μΙU/mL, respectively, under the same conditions. The results with commercially available transfection agents were about 9.9 ± 0.7 μIU/mL for insulin secretion, which is slightly lower than that from MPIP/RNPs-treated HEK293T cells. This difference must be attributed to the higher binding of dCas9 to the imprinted particles. The same stimulus protocol was then used and the release of insulin monitored over the long term (up to 30 days post-transfection), as shown in Figure 4d. During this time, cells continued to proliferate (requiring subculturing/splitting of the cells approximately every 3 days), indicating good cellular viability. Gene expression on RNA transfection has a typical half-life that varies from 1.5 to 18 h, depending on the delivery vehicle and method of introduction [36]. Surprisingly, the response following the stimulation was maintained for more than ten days, before decaying to half the original strength each week thereafter. However, synergistic effects from using multiple sgRNAs with dCas9-VP160 transfection system have been shown to sustain the activation of the INS gene for about 21 days post transfection [33].

**Figure 6:**
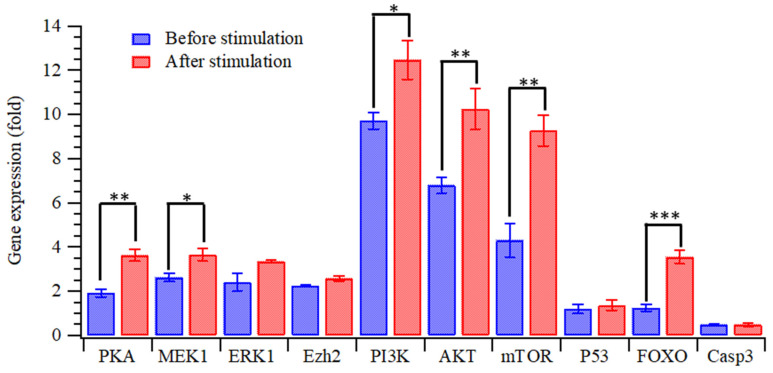
Relative gene expression of proteins of insulin-activated HEK-293T cells with MPIPs/RNPs before and after glucose stimulation. Many genes responsible for cellular proliferation are concomitantly upregulated with insulin. All experiments were carried out in triplicate, and data are expressed as means ± standard deviation; *: *p* < 0.05, **: *p* < 0.005, and ***: *p* < 0.0005.

Gene expression of HEK293T cells treated with RNPs on MPIPs, before and after glucose stimulation, can be found in the Supplementary Materials (S1.5). Figure 6 shows the expression levels of key proteins in the cellular signaling pathways (Figure S2) involving proliferation and insulin release, for transfected HEK 293T cells, before and after stimulation by glucose, respectively. Expression levels are normalized to the levels in untransfected cells. As shown in this figure, the expression of PI3K, Akt, and mTOR of the transfected HEK293T cells greatly exceeded that of the controls, even though there was no direct upregulation of these proteins [37]. The upregulation of insulin appears to concomitantly upregulate proliferation and insulin release proteins. These gene-expression levels were even higher after stimulation with glucose, and insulin-secretion-related genes, including PKA and MEK1, were then also activated [38]. The gene expression of FOXO was also activated; in pancreatic cells, FOXO is known to enhance β-cell secretory function (e.g., insulin secretion) [39].

## 4. Conclusions

The precise regulation of gene expression is of interest not only for treating diseases but also for the study of the development of tissues or organs. CRISPR systems have been recently used in gene editing; gene activation can be achieved using the CRISPR system with mutated Cas9 proteins [34]. In this work, mutated dCas9 proteins were conjugated with activators (like VPR), and crRNAs were synthesized for the activation of insulin expression. MPIPs were developed that bind the dCas9 and effectively deliver the CRISPR system into HEK293T cells. This construct effectively promoted the expression of insulin in this model system, and also resulted in the upregulation of proteins involved in insulin secretion and cell proliferation. We conclude that the direct delivery of ribonucleoprotein (RNP) with magnetic peptide-imprinted chitosan composite nanoparticles (MPIPs) is effective in HEK293T cells. This work holds promise for the development of a novel long-term insulin delivery system.

## Data Availability

The authors confirm that the data supporting the findings of this study are available within the article and its Supplementary Materials.

## Supplementary Materials

The following supporting information can be downloaded at: https://www.mdpi.com/article/10.3390/pharmaceutics15041311/s1, S1: Experiment [24,32,34,40]; Table S1: The sequence (5′-3′) of primers for GAPDH, INS, NEUROG3, NKX6.1, MAFA, PDX1; Table S2: The sequence (5′-3′) of primers for GAPDH, PI3k, AKT1, PKA, Casp3, mTOR, FOXO, P53, cAMP, EzH2, ERK1, MEK1; Figure S1: (a) The size distribution of the magnetic peptide-imprinted chitosan nanoparticles (MPIPs) with dCas9-VPR or RNPs at 20 nM. (b) MTT test of HEK293T cells treated with MPIP/RNPs; Figure S2: Schematic pathway of the glucose stimulation and gene expression.
