# Supplementary material for: Activation of Insulin Gene Expression via Transfection of a CRISPR/dCas9a System Using Magnetic Peptide-Imprinted Nanoparticles
Source: Pharmaceutics. 2023 Apr 21;15(4):1311. doi: 10.3390/pharmaceutics15041311 (PMC10144049; doi:10.3390/pharmaceutics15041311)
Supplement: Supplementary file 1 [file pharmaceutics-15-01311-s001.zip › pharmaceutics-2332520-supplementary.pdf]

pharmaceutics

Supplementary Materials: Activation of Insulin Gene Expression via Transfection of a CRISPR/dCas9a System Using Magnetic Peptide-imprinted Nanoparticles

Mei-Hwa Lee, James L. Thomas, Chien-Yu Lin, Yi-Chen Ethan Li and Hung-Yin Lin

S1. Experimental

S1.1 Reagents and Chemicals.

Iron (II) sulphate 7-hydrate (99.0%, #131362) was from Panreac (Barcelona, Spain). Chitosan (from shrimp shells, ≥75% (deacetylated), #C3646) and iron (III) chloride 6-hydrate (97%, #236489) were ordered from Sigma-Aldrich Chemical Company (St. Louis, MO). Acetic acid (ACS grade, #9511-05) and sodium hydroxide (#0405.1000) were purchased from J. T. Baker (Phillipsburg, NJ, USA). Peptide of Cas9 protein in the sequence of QLFVEQHKHYLDE was from Yao-Hong Biotechnology Inc. (HPLC grade, New Taipei City, Taiwan).

S1.2 Preparation and characterization of magnetic peptide-imprinted nanoparticles (MPIPs).

The preparation of magnetic peptide-imprinted nanoparticles (MPIPs) can be found elsewhere [24, 32]. Briefly, magnetic nanoparticles (MNPs) were added to the chitosan solution (chitosan/0.01 wt % acetic acid solution = 1.0 wt%) to a concentration of 0.1 mg/mL. The chitosan/MNP solution was then mixed with 0 or 1.0 μg/mL of peptide for magnetic peptide- and non-imprinted chitosan composite nanoparticles (MNIPs and MPIPs, respectively). The chitosan/peptide/MNPs suspension was dispersed in 10 mL deionized water at 4 ^{o}C followed by removal of the peptide from MPIPs by washing with 10 mL deionized (DI) water for 1 min and separating on a magnetic plate two times. All composite nanoparticles were equilibrated with deionized water overnight before use.

The nanoparticles including MNPs, MNIPs, MPIPs after peptide removal and rebound with dCas-VPR or RNPs (at 20nM) were monitored by a dynamic light scattering (DLS) particle sizer (90Plus, Brookhaven Instruments Co., New York, NY, USA). The magnetization of MNPs, MNIPs, MPIPs was monitored with a magnetic property measurement MPMS XL-7 system (Quantum Design, San Diego, CA, USA) at 298 K in ± 15,000 Gauss.

S1.3 Extraction of CRISPR/dCas9a proteins from transfected HEK 293T cells with MPIPs

Human embryonic kidney cells (HEK 293T) were purchased from ATCC (American Type Culture Collection). The culture medium for HEK 293T cells contains high glucose Dulbecco's Modified Eagle's Medium (DMEM, #11966) was from Gibco (Thermo Fisher Scientific Inc., Waltham, MA, USA), and without sodium pyruvate and sodium bicarbonate, 10% v/v of fetal bovine serum (FBS, #12003C) and 1% v/v of antibiotics (penicillin and streptomycin) were from Life Technologies Co. (Grand Island, NY, USA). Cas9 Plasmids (dCas9-p300-P2A, dCas9-SunTag-VP64-P2A, dCas9-VPR-P2A) were precious gifts from RNA technology platform and gene manipulation core, Academia Sinica, Taipei, Taiwan.

HEK293T $2x10^6$ cells were seeded in a 10 cm culture dish and kept at 37°C in 5% CO_2 for 24 hr. Ten micrograms plasmid DNA (1 mg/mL) was added into 500 μL jetPRIME buffer (jetPRIME, Polyplus Transfection®, Illkirch, France) and mixed by vortexing. 20 μL jetPRIME was added and briefly vortexed for 10s and then spun down briefly. The transfection mixture solution (jetPRIME, Polyplus Transfection®, Illkirch, France) was incubated for 10 min at room temperature. Finally, the transfection mix was added to the HEK

293T cells and then incubated for 24 h. Cells were then washed with 500 µL PBS and 5 µL protease inhibitor cocktail (HY-K0010-1, MedChemExpress LLC, Monmouth Junction, NJ, USA) RIPA lysis buffer (Biotechnology Grade, VWR International LLC, Radnor, PA, USA) were added into the culture dish and kept at 4 °C for 30 min. The mixture of cellular fluid and cells was collected into a test tube using a scraping cutter, and then treated with an ultrasonic bath for 10 s and 30 s rest for six time. The TPP centrifuge tubes were centrifuged at 11,000 ×g for 10 min. and the supernatant collected. Five hundred microgram of MPIPs were added into the collected liquid for 30 min, and the MPIPs were then collected on the wall using a magnet and removed. The CRISPR/Cas9 protein was released from MPIPs by washing the particles for 10 s with 200 L DI water, and the protein concentration was measured with ELISA kit [24, 32, 34].

S1.4 Immunohistochemistry of CRISPR/Cas9 and insulin proteins

HEK 293T 2x10^4 cells were seeded in a 24-well cell plate and kept at 37°C and 5% CO_2 for 24 hr. Each well was then washed with 400 µL PBS and the cells were fixed in 3.7 % formaldehyde in DI water for 10 min at room temperature. After further washes with 350 µL PBS in each well, cells were permeabilized with 1 % Triton X-100 for 10 min at room temperature, and then washed with 350 µL/well PBS, followed by blocking of nonspecific binding by washing in PBS supplemented with 5% BSA for 60 mins. Finally, 350 µL PBS was added in each well and cells were incubated overnight at 4 °C with (1) 1:500 mouse anti-Cas9 antibody (BioVision #A1069), (2) 1:800 rabbit anti-insulin antibody (Sino Biological, #101282-T02) or (3) 1:500 rabbit anti-PDX1 (#PA5-78024, Thermo Fisher) or anti-NKX6.1 (#PA5-23070, Thermo Fisher) and mouse anti-Ngn3 (#SAB3300089, Sigma-AldrichSigma-Aldrich) or anti-MAFA (#SAB4301757, Sigma-Aldrich) to BSA. The cells were then washed in PBS and labeled with 300 µL/well secondary antibody for 1 h at room temperature. After another wash in 250 µL PBS at room temperature, the cells were co-stained with the nuclear dye DAPI for 15 mins (Sigma). Finally, the cells were washed with PBS and examined with an inverted fluorescence microscope (CKX41, Olympus, Melville, NY, USA).

S1.5 Gene expression of HEK293T cells treated with RNPs on MPIPs, before and after glucose stimulation

Primers for activation of β cells and insulin release genes were listed in Tables S1 and S2 [34], respectively. 5' - 3' direction refers to the orientation of nucleotides of a single strand of DNA or RNA. The 5' and 3' specifically refer to the 5th and 3rd carbon atoms in the deoxyribose/ribose sugar ring. The total RNA extraction from (1) the HEK293T cells treated with various concentrations of RNPs adsorbed on MPIPs and (2) transfected cells before and after glucose stimulation was performed using the Nucleospin RNA, Mini kit for RNA purification (#740955.50 Macherey-Nagel). The concentration of cellular RNA was quantified by determining the absorbance maximum at the wavelength of 260 and 280 nm to optimum OD about 1.5 in a UV/Vis spectrometer (Lambda 40, PerkinElmer, Wellesley MA). Complementary DNA was obtained following a Magic RT Mastermix cDNA synthesis kit (BB-DBU-RT-100, Bio-genesis Technologies, lnc., Taipei, Taiwan) protocol. The real-time PCR was then performed with Fast SYBR™ Green Master Mix (#4385612, Thermo Fisher Scientific. Waltham, MA, USA) in a StepOne™ Real-Time PCR System (LS4376357, Applied Biosystems, Waltham, MA, USA). Relative gene expression was determined using a ΔΔCq method [40] and normalized to a reference gene (GAPDH) and to control (HEK293T).

S1.6 Data analysis

All experiments were carried out in triplicate, and data are expressed as means ± standard deviation and analyzed with Student’s t-test.

Table S1. The sequence (5′- 3′) of primers for GAPDH, INS, NEUROG3, NKX6.1, MAFA, PDX1.

mRNA	Forward/Reverse	Sequence (5′- 3′)
GAPDH	forward	CTTTTGCGTCGCCAG
	reverse	TTGATGGCAACAATATCCAC
INS	forward	CCATCAAGCAGATCACTG
	reverse	CACTAGGTAGAGAGCTTCC
Ngn-3	forward	CCCCATTCTCTCTTCTTTTC
	reverse	AGGCGTCATCCTTTCTAC
NKX6.1	forward	CCTGTACCCCTCATCAAG
	reverse	GACCTGACTCTCTGACATC
MAFA	forward	ACATATCTGTAACTCCTGGG
	reverse	CCACAAACAAAACGAAACAC
PDX1	forward	AAAACGTATGTGATTGGAGG
	reverse	CCAGACCTTGAAAAGAAGAC

Table S2. The sequence (5′- 3′) of primers for GAPDH, PI3k, AKT1, PKA, Casp3, mTOR, FOXO, P53, cAMP, EzH2, ERK1, MEK1.

mRNA	Forward/Reverse	Sequence (5′- 3′)
GAPDH	forward	CTTTTGCGTCGCCAG
	reverse	TTGATGGCAACAATATCCAC
PI3k	forward	CAGATTCTACGAATCATGGAG
	reverse	TCCTATTTTGTCACCAGTTG
AKT1	forward	AAGTACTCTTTCCAGACCC
	reverse	TTCTCCAGCTTGAGGTC
PKA	forward	CTGAGATTATCCTGAGCAAAG
	reverse	GCCATTTCATAGATAAGAACCC
Casp3	forward	AAAGCACTGGAATGACATC
	reverse	CGCATCAATTCCACAATTTC
mTOR	forward	GGAGGAGAAATTTGATCAGG
	reverse	GGGCAACAAATTAACGATTG
FOXO	forward	GTCAAGACAACGACACATAG
	reverse	AAACTAAAAGGGAGTTGGTC
P53	forward	AGGATTCACAGTCGGATATG
	reverse	GGAGGAAGAAGTTTCCATAAG
EzH2	forward	AAGAAATCTGAGAAGGGACC
	reverse	CTCTTTACTTCATCAGCTCG
ERK1	forward	TTCGAACATCAGACCTACTG
	reverse	TAGACATCTCTCATGGCTTC
MEK1	forward	GATTACATAGTCAACGAGCC
	reverse	CTTCAAATCTGCTCTCTCTG

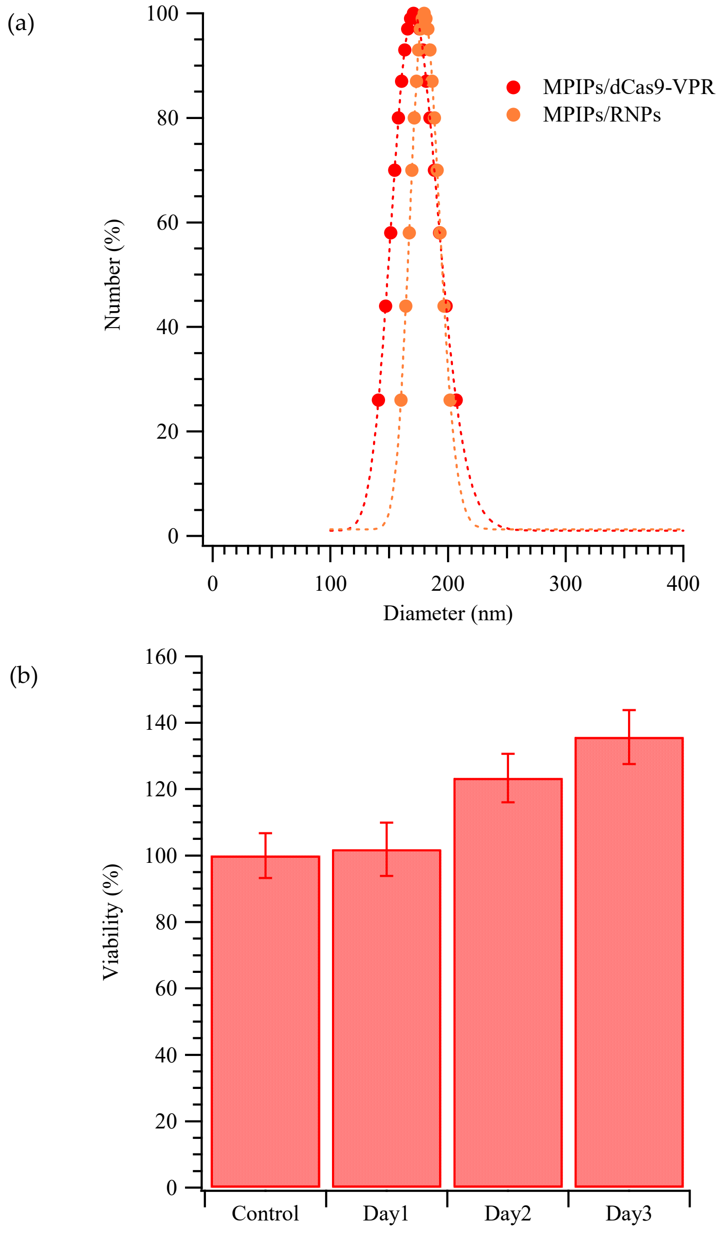

Figure S1. (**a**) The size distribution of the magnetic peptide-imprinted chitosan nanoparticles (MPIPs) with dCas9-VPR or RNPs at 20 nM. (**b**) MTT test of HEK293T cells treated with MPIP/RNPs.

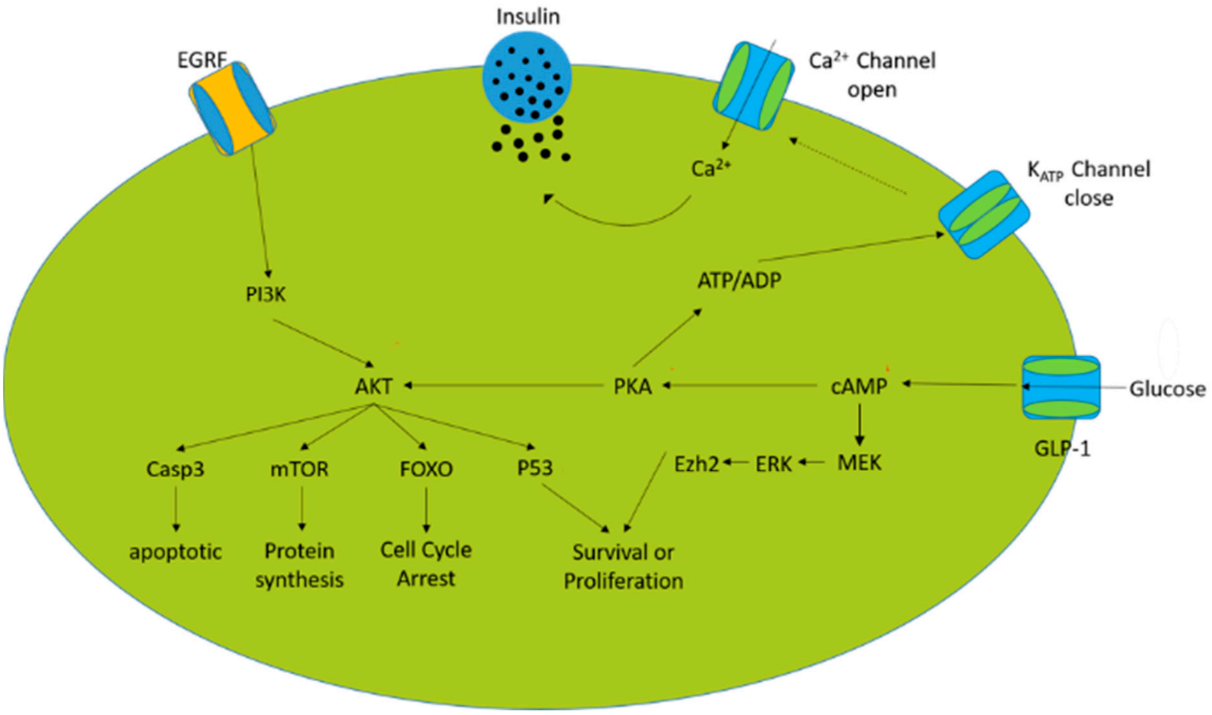

Figure S2. Schematic pathway of the glucose stimulation and gene expression.
